# Epimedium applied in the clinical treatment of osteoporosis patients with periodontitis

**DOI:** 10.1097/MD.0000000000040837

**Published:** 2024-12-13

**Authors:** Ying Guo, Xu Ge, Wei Wang, Rongrong Wang, Qianmin Chen, Hong Wang

**Affiliations:** a Department of stomatology, General Hospital of Shenzhen University, Shenzhen, China; b Dental Department, The University of Hong Kong-Shenzhen Hospital, Shenzhen, China.

**Keywords:** bone mineral density, epimedium, inflammatory markers, oral health, osteoporosis, periodontitis

## Abstract

Osteoporosis and periodontitis, prevalent in middle-aged and elderly populations, share common features of bone loss and chronic inflammation. This study explores the hypothesis that Epimedium, known for its bone-strengthening properties, may enhance the effectiveness of conventional osteoporosis treatment in patients with coexisting periodontitis. This retrospective study analyzed clinical data from 120 patients with osteoporosis and periodontitis, divided into 2 groups. The control group received calcium carbonate, vitamin D, and zoledronic acid (CC + VD + ZA) therapy, while the observation group received additional Epimedium flavonoid treatment. Outcomes assessed included changes in bone mineral density (BMD), bone metabolism markers (β-CTx, N-MID, CT, ALP), periodontal indices (PD, AL, SBI, PLI), and inflammatory markers in gingival crevicular fluid (GCF) before and 6 months posttreatment. Compared to the control group, the observation group showed significantly greater increases in lumbar spine and proximal femur BMD and reductions in BM markers (*P* < .05). Periodontal health metrics (PD, AL, SBI, PLI) and GCF inflammatory markers (TNF-α, IL-1β, IL-6, IL-8, hs-CRP, ICAM-1, HMGB1, PGE2) were markedly improved in the observation group, correlating with enhanced total effective rates (TER) for osteoporosis (95.0%) and periodontitis (91.7%) and a reduced adverse event rate (AER). Epimedium shows promise as an adjunctive therapy in patients with osteoporosis and periodontitis, contributing to improved BMD, reduced inflammation, and enhanced periodontal health, suggesting its potential for broader clinical application in managing these coexisting conditions.

## 1. Introduction

Osteoporosis is a widespread degenerative bone disease that primarily affects middle-aged and elderly individuals, characterized by reduced bone mineral density (BMD), compromised bone structure, and increased bone fragility, which elevates fracture risk and significantly impacts patient health.^[1]^ Periodontitis, a prevalent chronic inflammatory disease in the oral cavity, also affects older populations, leading to gum inflammation, tooth mobility, and alveolar bone resorption, thereby impacting patients’ quality of life (QOL).^[2]^ Notably, both osteoporosis and periodontitis share key features of progressive bone loss and susceptibility to inflammatory processes, which are influenced by age, hormonal changes, smoking, and deficiencies in calcium and vitamin D.^[3,4]^ This connection points to a potentially bidirectional relationship, where bone resorption processes in osteoporosis may be exacerbated by periodontal inflammation, and vice versa.^[5]^

Evidence suggests that BMD in the maxillofacial region is often compromised in osteoporosis patients, which may accelerate periodontal tissue degradation and further underscore the link between these 2 conditions.^[6]^ Given their overlapping pathophysiology, treatments that simultaneously target bone and periodontal health may hold promise in improving outcomes for patients suffering from both diseases. Epimedium, a traditional Chinese herb rich in flavonoids, has shown potential in promoting bone cell growth, enhancing osteoblast differentiation, and inhibiting osteoclast activity, contributing to improved bone health.^[7–9]^ Experimental models of osteoporosis indicate that Epimedium can increase BMD, improve trabecular structure, and mitigate bone resorption, suggesting it may be beneficial in preventing or treating osteoporosis.^[10]^ However, despite its demonstrated osteogenic and anti-inflammatory effects in preclinical studies, the clinical impact of Epimedium on osteoporosis patients with concurrent periodontitis remains unclear.^[11]^

This study hypothesizes that Epimedium, when used as an adjunct to standard osteoporosis treatment, will enhance BMD and simultaneously reduce periodontal inflammation in patients with coexisting osteoporosis and periodontitis. To evaluate this, we retrospectively analyzed clinical data from patients receiving either conventional osteoporosis therapy or combined therapy with Epimedium, focusing on BMD, periodontal health, and inflammatory markers. This investigation seeks to provide new insights into managing osteoporosis with concurrent periodontal disease and supports the potential of Epimedium as a complementary therapy for these coexisting conditions.

## 2. Objects and methods

### 2.1. Study design

This study was approved by the Ethics Committee of the General Hospital of Shenzhen University. This study was a retrospective analysis conducted to evaluate the clinical efficacy of total Epimedium flavones (TEF) as an adjuvant to standard osteoporosis and periodontitis treatment. Patients were divided into 2 groups based on their treatment regimens: the control group, receiving conventional therapy with calcium carbonate (CC), vitamin D (VD), and zoledronic acid (ZA), and the observation group, receiving the same conventional therapy in combination with TEF capsules. As noted in the Introduction, TEF contains both Epimedium and its active compounds, including flavonoids, lignans, alkaloids, and volatile oils. Given this, the study did not separate Epimedium from flavones, as the research focus was on the combined effect of these components as delivered in the TEF formulation.

### 2.2. Study participants

**Figure 1.**
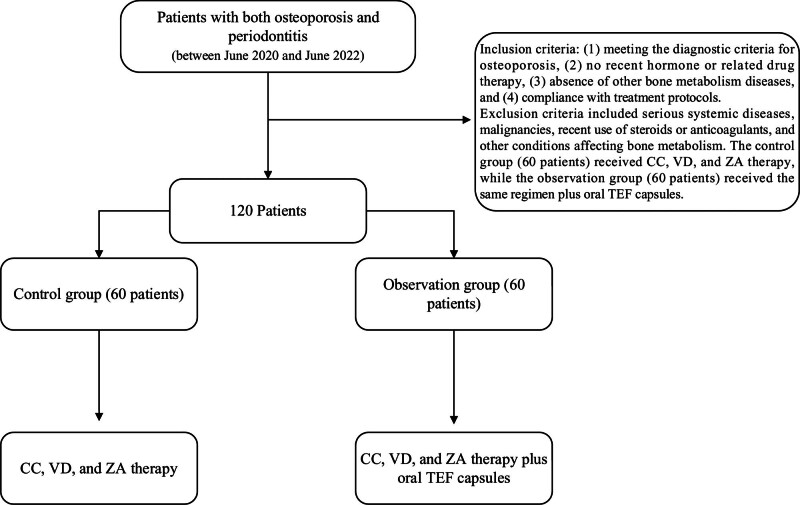
Flow chart.

Clinical data were retrospectively collected from 120 patients with both osteoporosis and periodontitis who received treatment between June 2020 and June 2022 at Shenzhen University General Hospital and Shenzhen Hospital of the University of Hong Kong. Patients were enrolled based on the following inclusion criteria: meeting the diagnostic criteria for osteoporosis; no recent hormone or related drug therapy; absence of other BM diseases; and compliance with treatment protocols. Exclusion criteria included serious systemic diseases, malignancies, recent use of steroids or anticoagulants, and other conditions affecting BM. The control group (60 patients) received CC, VD, and ZA therapy, while the observation group (60 patients) received the same regimen plus oral TEF capsules. The process is shown in Figure 1.

### 2.3. Treatment protocol

All patients received standard periodontal care, including subgingival scaling and the insertion of tinidazole film (0.5 mg per piece) into the periodontal pockets to control local infection. Patients in the control group were administered CC D3 tablets (Wyeth Pharmaceutical Co., LTD., 600 mg/day, specification: 600 mg * 30 tablets, batch number: National Drug Approval (NDA) H10950029) and intravenous zoledronic acid (ZA, Yangzijiang Pharmaceutical Group Sichuan Hairong Pharmaceutical Co., LTD., 5 mg in 100 mL solution, specification: 5 mL: 4 mg, batch number: NDA H20123153), infused over 15 minutes with hydration before and after administration. In addition to the conventional therapy, patients in the observation group were given 2 total Epimedium flavone (TEF) capsules (Jiangsu Kangyuan Sunshine Pharmaceutical Co., LTD., 0.35 g per capsule containing 309 mg Epimedium flavone extract, batch number: Z20140012) 3 times per day, with a treatment course extending over 24 weeks.

### 2.4. Outcome measures and data collection

1. Bone Mineral Density (BMD): BMD was assessed at the lumbar spine (L1–L4) and proximal femur using DXA scanning to evaluate bone strength and osteoporosis severity.
2. Bone Metabolism Markers: Serum levels of β-CTx (indicating bone resorption), N-MID (bone formation marker), Calcitonin (osteoclast inhibitor), and ALP (bone remodeling enzyme) were measured to monitor BM.
3. Periodontal Health Indicators: Periodontal health was evaluated using Periodontal Depth (PD), Attachment Loss (AL), Sulcus Bleeding Index (SBI), and Plaque Index (PLI) to gauge inflammation, tissue damage, and oral hygiene.
4. Inflammatory Markers in Gingival Crevicular Fluid (GCF): Markers including TNF-α, IL-1β, IL-6, IL-8, hs-CRP, ICAM-1, HMGB1, and PGE2 were measured in GCF to assess the level of periodontal inflammation.
5. Scale Scores: Patient outcomes were assessed through the Self-Efficacy Scale for Self-Care (SESS), Geriatric Oral Health Assessment Index (GOHAI), and Barthel Index to evaluate oral health self-care, impact on QOL, and daily functional ability.

### 2.5. Statistical analysis

Data were analyzed using SPSS 19.0 software. Descriptive statistics were presented as mean ± standard deviation (SD) for continuous variables and as frequencies for categorical data. Group comparisons were conducted using *t*-tests for continuous variables and chi-square tests for categorical data. To account for multiple comparisons, we applied the Bonferroni correction by multiplying the *P*-value by the number of comparisons for each test. All *P*-values reported in the results are Bonferroni-adjusted, with a significance threshold of < .05, ensuring clarity and consistency across analyses. This adjustment confirmed the combined benefit of TEF as an adjuvant therapy, enhancing BMD and periodontal health in patients with osteoporosis and periodontitis.

## 3. Results

### 3.1. BMD changes in osteoporosis patients with periodontitis before and after treatment

**Figure 2.**
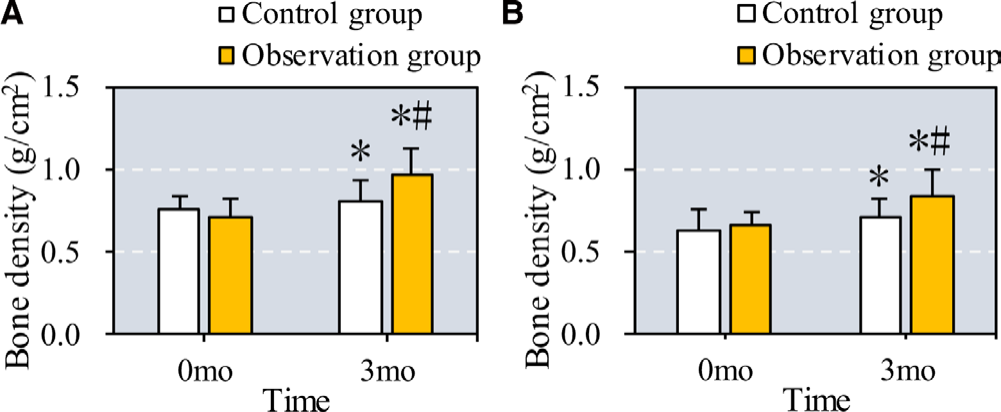
Comparison of BMD of patients before and after they were treated with different methods. Note: A and B exhibit the comparison of BMD in L1 to L4 lumbar vertebra and proximal femur, respectively, before and after the patients were treated with different methods. * and # suggest the difference exhibited a significance with *P* < .05 based on the values before the treatment and in the Ctrl group, respectively. BMD = bone mineral density.

The study assessed changes in BMD in the lumbar spine (L1–L4) and proximal femur before and after treatment in patients with concurrent osteoporosis and periodontitis. As shown in Figure 2, BMD significantly increased in both lumbar L1 to L4 segments and the proximal femur in both the control group (treated with CC + VD + ZA) and the observation group (treated with CC + VD + ZA plus TEF) after 6 months (*P* = .03 for lumbar spine, *P* = .04 for proximal femur in the control group). Furthermore, compared to the control group, patients in the observation group demonstrated a more substantial BMD increase in both regions (*P* = .01 for lumbar spine, *P* = .02 for proximal femur) after 6 months of treatment, indicating an enhanced effect from the addition of TEF.

### 3.2. Comparison of bone metabolism (BM) indexes in osteoporosis patients with periodontitis before and after treatment

**Figure 3.**
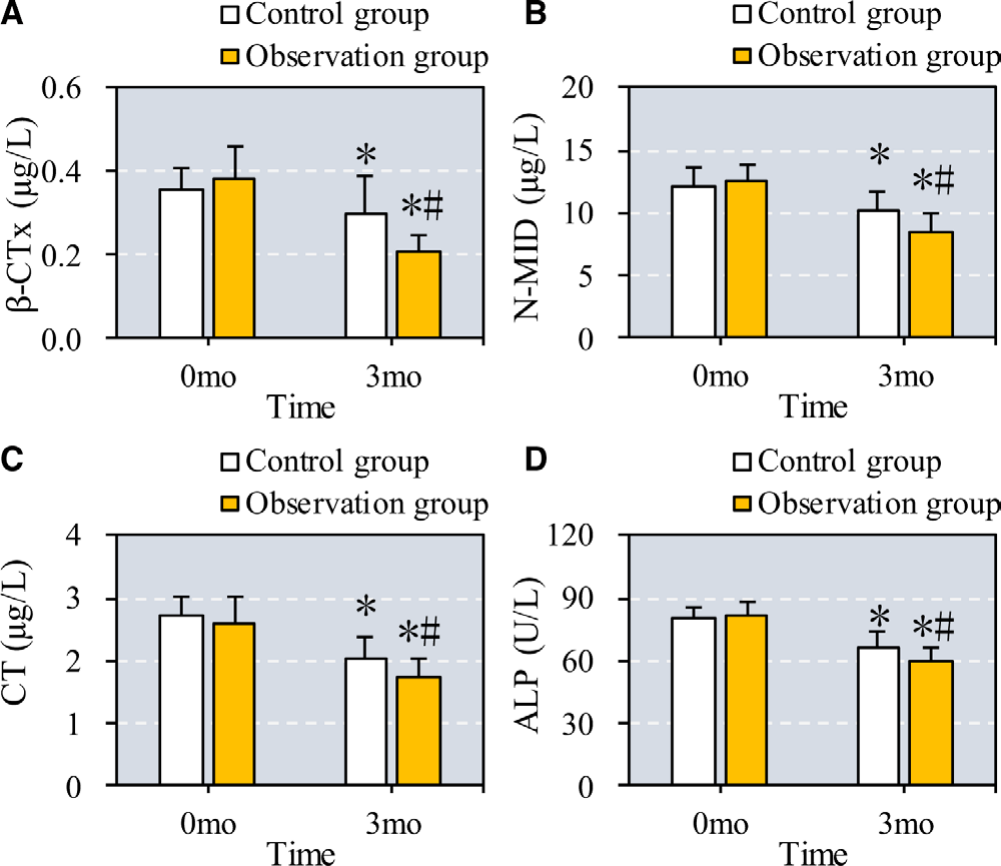
Comparison of BM indexes before and after the patients were treated with different drugs. Note: A to D show the comparison of β-CTx, N-MID, CT, and ALP of patients from different groups. * and # suggest the difference exhibited a significance with *P* < .05 based on the values before the treatment and in the Ctrl group, respectively. ALP = alkaline phosphatase, BM = bone metabolism, CT = calcitonin, N-MID = N-terminal osteocalcin, β-CTx = β-crosslaps.

The study examined changes in BM indexes, specifically β-Crosslaps (β-CTx), N-terminal osteocalcin (N-MID), calcitonin (CT), and alkaline phosphatase (ALP), in patients with osteoporosis and periodontitis before and after treatment (as shown in Fig. 3). After 6 months, both the control group (treated with CC + VD + ZA) and the observation group (treated with CC + VD + ZA plus TEF) showed significant decreases in β-CTx, N-MID, CT, and ALP levels compared to baseline (*P* = .03 for β-CTx, *P* = .04 for N-MID, *P* = .02 for CT, and *P* = .03 for ALP in the control group). Additionally, BM indexes in the observation group were significantly lower than those in the control group after treatment, indicating enhanced effectiveness with TEF (*P* = .01 for β-CTx, *P* = .02 for N-MID, *P* = .01 for CT, and *P* = .02 for ALP).

### 3.3. Periodontal indexes in osteoporosis patients with periodontitis before and after treatment

**Figure 4.**
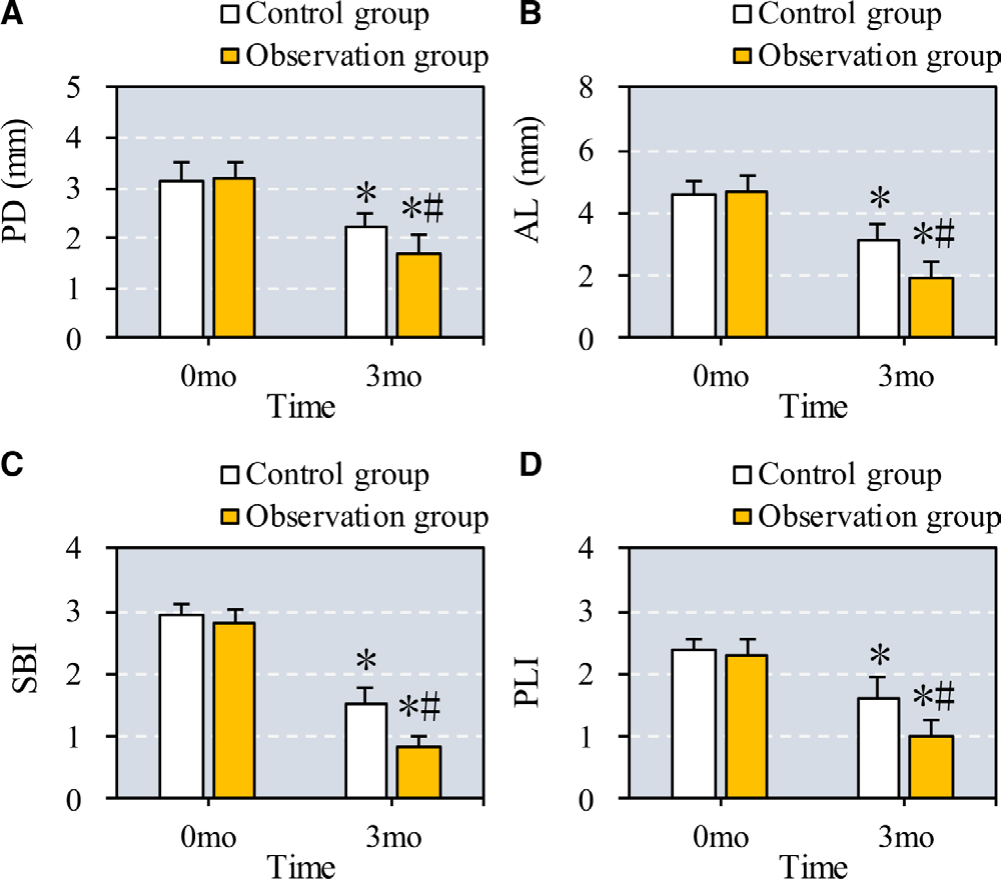
Comparison of periodontal indexes before and after the patients from different groups were treated. Note: A to D show the comparison PD, AL, SBI, and PLI, respectively. * and # suggest the difference exhibited a significance with *P* < .05 based on the values before the treatment and in the Ctrl group, respectively. AL = attachment loss, PD = periodontal depth, PLI = plaque index, SBI = sulcus bleeding index.

The study assessed changes in periodontal indexes, including periodontal depth (PD), attachment loss (AL), sulcus bleeding index (SBI), and plaque index (PLI), in patients with osteoporosis and periodontitis before and after treatment. As shown in Figure 4, both the control group (CC + VD + ZA) and the observation group (CC + VD + ZA plus TEF) demonstrated significant reductions in PD, AL, SBI, and PLI after 6 months of treatment (*P* = .03 for PD, *P* = .04 for AL, *P* = .02 for SBI, and *P* = .03 for PLI in the control group). The observation group exhibited even greater decreases in these periodontal indexes compared to the control group, highlighting the additional benefits of TEF (*P* = .01 for PD, *P* = .02 for AL, *P* = .01 for SBI, and *P* = .02 for PLI).

### 3.4. Inflammatory factors in GCF in patients with osteoporosis and periodontitis

**Figure 5.**
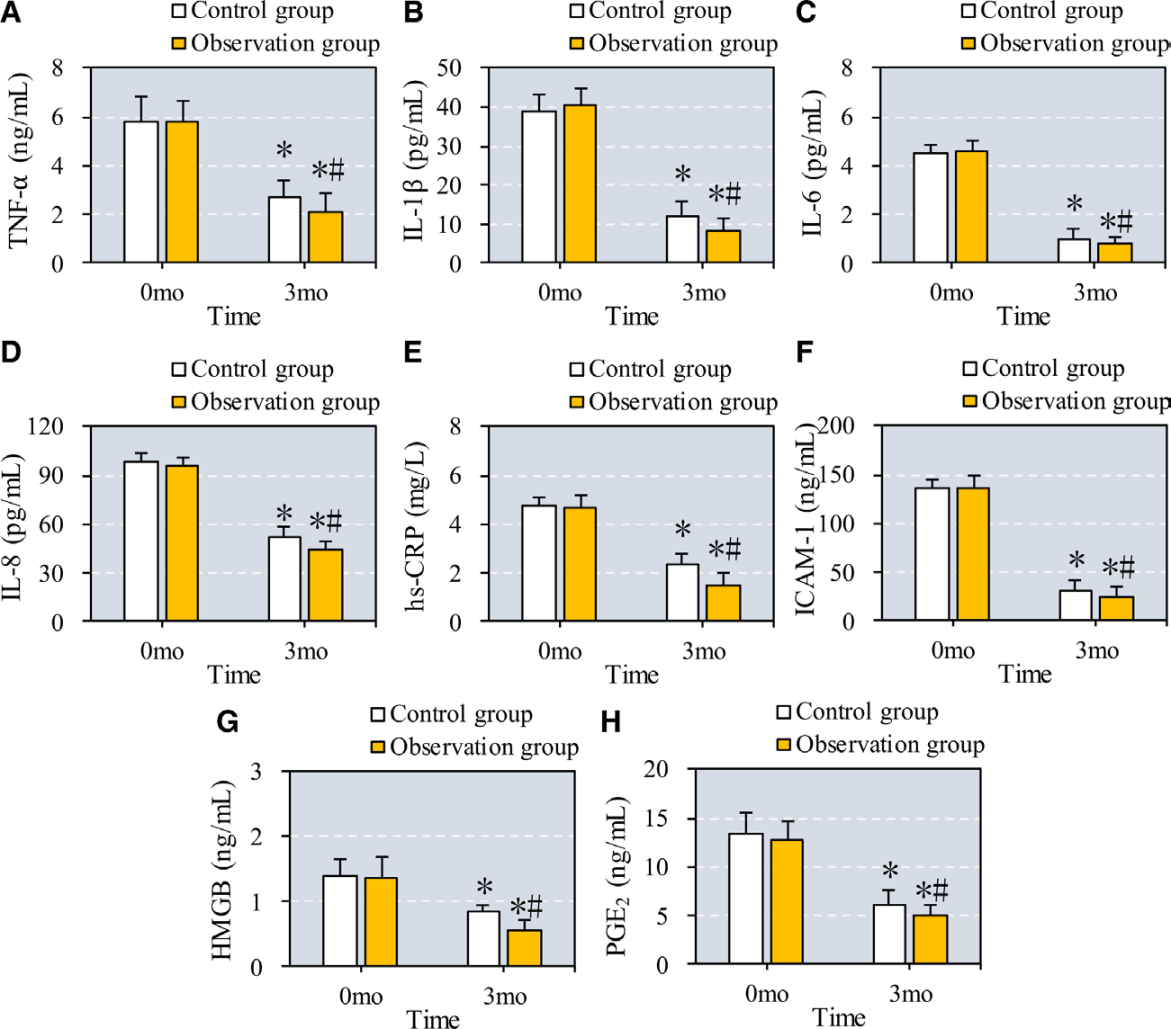
Comparison of GCF inflammatory factors before and after the patients were treated. Note: A to H illustrate the comparison of TNF-α, IL-1β, IL-6, IL-8, hs-CRP, ICAM-1, HMGB1, and PGE2 in the GCF of patients before and after they were treated with different methods. * and # suggest the difference exhibited a significance with *P* < .05 based on the values before the treatment and in the Ctrl group, respectively. GCF = gingival crevicular fluid, HMGB1 = high mobility group protein 1, hs-CRP = hypersensitive C-reactive protein, ICAM-1 = intercellular adhesion molecule-1, TNF-α = tumor necrosis factor-α, PGE2 = prostaglandin E2.

This study evaluated changes in inflammatory markers within GCF, including TNF-α, IL-1β, IL-6, IL-8, hs-CRP, ICAM-1, HMGB1, and PGE2, before and after treatment in patients with osteoporosis and periodontitis. As presented in Figure 5, levels of all markers (TNF-α, IL-1β, IL-6, IL-8, hs-CRP, ICAM-1, HMGB1, and PGE2) significantly decreased after 6 months in both the control group (CC + VD + ZA) and the observation group (CC + VD + ZA plus TEF) when compared to baseline (*P* = .04 for TNF-α, *P* = .03 for IL-1β, *P* = .02 for IL-6, *P* = .03 for IL-8, *P* = .02 for hs-CRP, *P* = .03 for ICAM-1, *P* = .04 for HMGB1, and *P* = .02 for PGE2 in the control group). The observation group demonstrated significantly greater reductions in these inflammatory markers compared to the control group, underscoring the added anti-inflammatory benefit of TEF (*P* = .01 for TNF-α, *P* = .02 for IL-1β, *P* = .01 for IL-6, *P* = .01 for IL-8, *P* = .02 for hs-CRP, *P* = .01 for ICAM-1, *P* = .03 for HMGB1, and *P* = .02 for PGE2).

### 3.5. Comparison of scale scores in patients with osteoporosis and periodontitis

**Figure 6.**
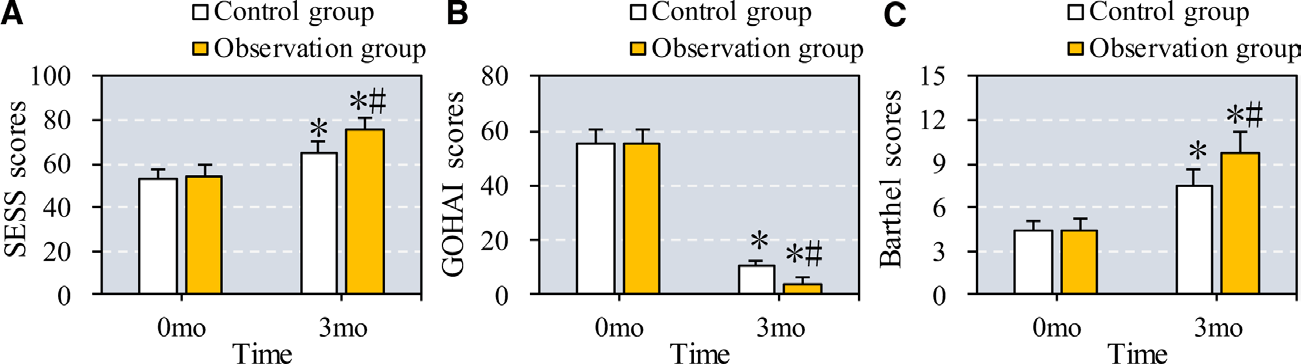
Comparison of scores of relevant scales of patients before and after they were treated. Note: A to C display the score comparison SESS, GOHAI, and Barthel scales, respectively. * and # suggest the difference exhibited a significance with *P* < .05 based on the values before the treatment and in the Ctrl group, respectively. GOHAI = geriatric oral health assessment index, SESS = self-efficacy scale for self-care.

The study assessed changes in the Self-Efficacy Scale for Self-Care (SESS), GOHAI, and Barthel Index in patients with osteoporosis and periodontitis before and after treatment. As shown in Figure 6, both the control group (CC + VD + ZA) and the observation group (CC + VD + ZA plus TEF) demonstrated significant increases in SESS and Barthel scores, alongside significant decreases in GOHAI scores after 6 months (*P* = .03 for SESS, *P* = .02 for Barthel, and *P* = .04 for GOHAI in the control group). The observation group exhibited a more substantial increase in SESS and Barthel scores, as well as a more pronounced decrease in GOHAI scores, indicating an enhanced improvement in self-care efficacy, daily function, and oral health with the addition of TEF (*P* = .01 for SESS, *P* = .02 for Barthel, and *P* = .01 for GOHAI compared to the control group).

### 3.6. Therapeutic effects in patients

Table 1 presents the therapeutic outcomes of different treatment methods for patients with osteoporosis and periodontitis. In the control group (CC + VD + ZA), 30 patients showed an “obviously effective” response, 18 were “effective,” and 12 were “ineffective.” In contrast, in the observation group (CC + VD + ZA plus TEF), 33 patients showed an “obviously effective” response, 24 were “effective,” and only 3 were “ineffective.” The total effective rates (TER) were 80.0% in the control group and 95.0% in the observation group, indicating a significantly greater therapeutic effect in the observation group (*P* = .03). The therapeutic effects of different treatments on symptoms associated with osteoporosis and periodontitis are summarized in Table 2. In the control group (CC + VD + ZA), 30 patients experienced an “obviously effective” outcome, 15 were “effective,” and 15 were “ineffective.” In the observation group (CC + VD + ZA plus TEF), 23 patients showed an “obviously effective” response, 14 were “effective,” and only 3 were “ineffective.” The total effective rate (TER) was significantly higher in the observation group (91.7%) compared to the control group (75.0%), indicating a greater therapeutic benefit with the addition of TEF (*P* = .02).

**Table 1:** Evaluation of the effect of osteoporosis patients in different groups.

Group	Cases	Obviously effective	Effective	Ineffective	TER
Ctrl group	60	30 (50.0)	18 (30.0)	12 (20.0)	48 (80.0)
Obs group	60	33 (55.0)	24 (40.0)	3 (5.0)	57 (95.0)

Abbreviation: TER = total effective rate.

**Table 2:** The symptom treatment results to periodontitis.

Group	Cases	Obviously effective	Effective	Ineffective	TER
Ctrl group	60	30 (50.0)	15 (25.0)	15 (25.0)	45 (75.0)
Obs group	60	33 (55.0)	22 (36.7)	5 (8.3)	37 (91.7)

Abbreviation: TER = total effective rate.

### 3.7. Adverse reactions in patients after treatment

**Figure 7.**
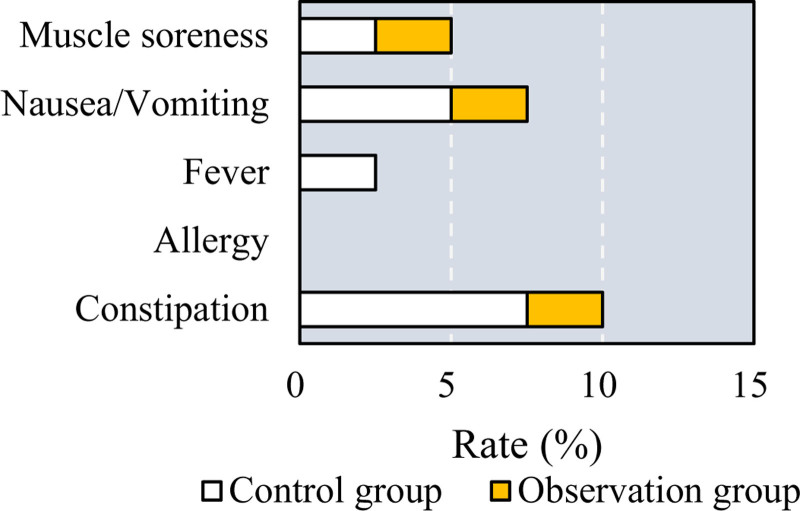
Comparison of ADR of patients after they were treated with different methods.

Adverse drug reactions (ADR) were recorded and compared between patients treated with different methods for osteoporosis and periodontitis. As shown in Figure 7, in the control group, there were 5 cases of constipation, 0 cases of allergy, 1 case of fever, 3 cases of nausea and vomiting, and 1 case of muscle soreness, resulting in a total ADR rate of 16.7%. In the observation group (CC + VD + ZA plus TEF), there were 2 cases of constipation, 0 cases of allergy, 0 cases of fever, 2 cases of nausea and vomiting, and 2 cases of muscle soreness, with a total ADR rate of 10.0%. The ADR rate in the observation group was significantly lower than in the control group (*P* = .04), indicating an improved safety profile with the addition of TEF.

## 4. Discussion

Osteoporosis is a prevalent degenerative bone disease among the elderly, characterized by systemic bone mass reduction, disrupted bone structure, and increased fragility, all of which heighten the risk of fractures.^[12–15]^ Due to diminished blood supply in elderly bones, levels of intraosseous matrix and bone minerals decrease, inhibiting bone formation and accelerating bone loss.^[16]^ Periodontitis, another common condition in this demographic, is a chronic inflammatory disease affecting oral health. Studies indicate a strong association between osteoporosis and periodontitis, with osteoporosis increasing susceptibility to periodontal tissue damage.^[17]^ In a cross-sectional study, Hong et al^[18]^ found that osteoporosis nearly doubled the risk of periodontitis and increased fracture susceptibility in periodontitis patients, underscoring the need for integrated treatment approaches. Our study highlights the importance of identifying treatments that can address both conditions concurrently.

Epimedium, a traditional Chinese medicinal herb, is widely recognized for its bone-strengthening properties. Previous studies have shown that Epimedium’s flavonoid compounds inhibit osteoclast differentiation, reduce bone resorption, and support bone health.^[19,20]^ Icariin, a primary active component in Epimedium, has been shown to enhance trabecular thickness, increase bone density, and improve the inflammatory profile in animal models.^[21]^ By promoting osteogenic differentiation of bone marrow stromal cells (BMSCs) and inhibiting adipogenic differentiation, Icariin can increase alkaline phosphatase (ALP) activity and enhance bone formation, indicating its potential as a therapeutic agent in both systemic osteoporosis and localized periodontal defects.^[22]^ This study evaluated the clinical efficacy and safety of Epimedium as an adjunct therapy in patients with osteoporosis and periodontitis.

Our findings reveal that the combination of conventional therapy with Epimedium significantly improves BMD and reduces BM markers, such as β-CTx, N-MID, CT, and ALP, compared to conventional treatment alone. This aligns with previous meta-analyses suggesting that Epimedium enhances BMD and reduces pain and ALP levels in osteoporosis patients.^[23,24]^ These results support the use of Epimedium as an effective adjunct therapy for osteoporosis and periodontitis, with improvements in both BMD and systemic BM markers.

In terms of periodontal health, patients treated with Epimedium showed significant improvements in periodontal indexes, including PD, AL, SBI, and PLI, likely due to the herb’s anti-inflammatory properties, which mitigate alveolar bone resorption. Chronic periodontitis involves persistent inflammation, where microorganisms and inflammatory mediators from the periodontal pocket enter systemic circulation, activating immune cells and increasing cytokine release.^[25,26]^ TNF-α and other inflammatory factors, such as IL-1β, IL-6, and IL-8, are central to this response, promoting bone resorption and inhibiting periodontal tissue repair.^[27–30]^ In our study, Epimedium significantly reduced GCF levels of TNF-α, IL-1β, IL-6, IL-8, hs-CRP, ICAM-1, HMGB1, and PGE2, indicating a reduction in inflammatory response and improved periodontal outcomes, with a TER of 91.7%.^[31–35]^

Our findings suggest that Epimedium is beneficial not only for improving BMD but also for managing periodontal inflammation, supporting its dual role in treating osteoporosis and periodontitis. The observed improvements in SESS and Barthel scores, along with reduced GOHAI scores, highlight its potential to enhance oral health self-efficacy, daily functioning, and overall QOL in patients. Furthermore, the safety profile of Epimedium is promising, with only mild adverse reactions reported, suggesting its suitability for long-term use as an adjunct therapy.

### 4.1. Limitations

We acknowledge several limitations in this study that may affect the generalizability and precision of its findings. Firstly, although data were collected from Shenzhen University General Hospital and Shenzhen Hospital of the University of Hong Kong to broaden the study’s scope, the sample may still lack sufficient geographical diversity. This may restrict the applicability of the results to other populations. We have highlighted this limitation in the discussion and suggest that future studies incorporate a multi-center design with more diverse geographic representation to improve the generalizability of the findings.

Secondly, while lifestyle factors, including diet, exercise frequency, and smoking status, are known to significantly impact BMD and periodontal health, collecting and quantifying such data can be challenging. Although controlling for these factors was difficult in our study, we recognize that they could act as potential confounders affecting the outcomes. This limitation has been acknowledged in the discussion, and we recommend that future research consider more rigorous documentation and control of lifestyle-related variables to reduce confounding effects and yield a clearer understanding of Epimedium’s clinical efficacy.

## 5. Conclusion

This study demonstrates that Epimedium, used as an adjuvant therapy for osteoporosis combined with periodontitis, effectively enhances BMD and reduces inflammatory markers in BM, periodontal tissues, and GCF. Additionally, Epimedium adjuvant therapy improves clinical symptoms, oral health, and the self-efficacy of oral health care, while also enhancing daily living abilities, with a favorable safety profile. However, this study primarily focused on the clinical effects of total flavone capsules of Epimedium and did not explore the specific mechanisms of action for each component. Further research is warranted to investigate the distinct mechanisms of Epimedium’s active compounds in treating osteoporosis and periodontitis. Overall, the findings support the potential of total flavone capsules of Epimedium as a valuable adjunct therapy in improving the prognosis of patients with osteoporosis and periodontitis.

## Author contributions

**Conceptualization:** Ying Guo, Xu Ge, Wei Wang, Rongrong Wang, Qianmin Chen, Hong Wang.

**Data curation:** Ying Guo, Xu Ge, Wei Wang, Rongrong Wang, Qianmin Chen, Hong Wang.

**Formal analysis:** Ying Guo, Xu Ge, Wei Wang, Rongrong Wang, Qianmin Chen, Hong Wang.

**Investigation:** Ying Guo, Xu Ge, Wei Wang, Rongrong Wang, Qianmin Chen, Hong Wang.

**Methodology:** Ying Guo, Xu Ge, Wei Wang, Rongrong Wang, Qianmin Chen, Hong Wang.

**Visualization:** Ying Guo, Hong Wang.

**Writing – original draft:** Ying Guo, Xu Ge, Hong Wang.

**Writing – review & editing:** Ying Guo, Xu Ge, Hong Wang.
